# Detecting early kidney damage in horses with colic by measuring matrix metalloproteinase -9 and -2, other enzymes, urinary glucose and total proteins

**DOI:** 10.1186/1751-0147-49-4

**Published:** 2007-01-23

**Authors:** Bela M Arosalo, Marja Raekallio, Minna Rajamäki, Elina Holopainen, Tuulia Kastevaara, Hanna Salonen, Satu Sankari

**Affiliations:** 1 Department of Equine and Small Animal Medicine, Pharmacology and Toxicology, Faculty of Veterinary Medicine, P.O. Box 57, 00014 University of Helsinki, Finland

## Abstract

**Background:**

The aim of the study was to investigate urine matrix metalloproteinase (MMP-2 and -9) activity, alkaline phosphatase/creatinine (U-AP/Cr) and gamma-glutamyl-transpeptidase/creatinine (U-GGT/Cr) ratios, glucose concentration, and urine protein/creatinine (U-Prot/Cr) ratio and to compare data with plasma MMP-2 and -9 activity, cystatin-C and creatinine concentrations in colic horses and healthy controls. Horses with surgical colic (n = 5) were compared to healthy stallions (n = 7) that came for castration. Blood and urine samples were collected. MMP gelatinolytic activity was measured by zymography.

**Results:**

We found out that horses with colic had significantly higher urinary MMP-9 complex and proMMP-9 activities than horses in the control group. Colic horses also had higher plasma MMP-2 activity than the control horses. Serum creatinine, although within reference range, was significantly higher in the colic horses than in the control group. There was no significant increase in urinary alkaline phosphatase, gamma-glutamyltranspeptidase or total proteins in the colic horses compared to the control group. A human cystatin-C test (Dako Cytomation latex immunoassay^® ^based on turbidimetry) did not cross react with equine cystatin-C.

**Conclusion:**

The results indicate that plasma MMP-2 may play a role in the pathogenesis of equine colic and urinary MMP-9 in equine kidney damage.

## Background

Usually the detection of kidney damage can be made at a relatively late stage by measuring serum creatinine and urea concentrations because at the time of abnormal values 75% of nephrons are already damaged [1]. However, depending on the cause and extend of damage, kidney has ability to recover [2-5]. There is a need for tests that can measure the early stages of kidney damage when there is still a chance to prevent further damage. In the studies made with laboratory animals the release of endotoxins can affect kidney function [6,7]. In severe equine colic the release of endotoxins is presumed to happen, but to our knowledge there are no studies on effects of endotoxins on equine kidneys. The routine use of nephrotoxic drugs like gentamicin, and non-steroidal anti-inflammatory drugs preoperatively can further increase risk of damage to the kidneys. For instance, even though gentamicin nephrotoxicity appears to be related to high or low levels of the drug, it can occur at recommended doses. Additionally, dehydration, and other drugs can affect the toxicity of gentamicin. Moreover, a deterioration in renal function may become evident for the first time or may progress after the drug has been discontinued [8]. Gunson et al. [9] reported that horses with both water deprivation and concurrent administration of the non-steroidal drug developed acute necrosis of the renal papilla, whereas the groups of horses that received either non-steroidal drug or water deprivation alone did not develop kidney damage.

A recent extensive review of all studies examining the clinical utility of cystatin-C in humans concluded that serum cystatin-C seems to perform at least as well as s-creatinine as an indicator of renal function, and is probably more sensitive than s-creatinine to small changes in GFR [10,11]. In dogs cystatin-C concentration seemed to correlate with s-creatinine concentrations in chronic renal failure, although that correlation was not as good with volume-depleted dogs [12]. No data exist regarding its use as a marker of GFR in horses.

Matrix metalloproteinases (MMPs) were characterized initially by their extensive ability to degrade extra-cellular matrix proteins. Some of the MMPs are involved in normal cell apoptosis and some are triggered by pathological processes like inflammation, ischemia, necrosis, tumor invasiveness, etc. Tashiro et al. [13] showed that patients with type-2 diabetic nephropathy and microalbuminuria had increased activity of urinary MMP-9 when s-creatinine and BUN concentrations were still normal. Basile et al. [14] showed that in rats kidney tissue MMP-2 activity increased two- to threefold in days 1 to 3 after ischemic acute renal failure and MMP-9 activity increased two- to threefold in days 2 to 3. To our knowledge, MMP activities in urine have not been measured in equine patients.

In humans, urine protein content is determined by collecting urine during a 24 hour period. With animals this is very unpractical and, therefore, urine protein concentration can be related to urine creatinine (U-Prot/Cr). Proteinuria may occur with glomerular disease, pyuria or, transiently, follow exercise [15]. In rhabdomyolysis large amounts of myoglobin can also be passed into urine. Currently, for adult horses there are no reported reference values for U-Prot/Cr ratio in the literature.

Two enzymes often examined in urine when kidney damage is suspected are alkaline phosphatase (AP), with its several isoenzymes, and gamma-glutamyltranspeptidase (GGT). In several studies with various species, an early increase in urine enzyme activity in AP and GGT has been found in the assessment of sub-clinical nephrotoxicity in tubular cells. They preceded the detection of azotemia and isosthenuria by several days in acute renal failure and by up to several years in slowly developing chronic renal diseases. [16-21] In healthy horses, there is no significant diurnal variation in the activity of U-GGT and -AP within the individual. Neither sex nor age seems to influence U-GGT activity. [22]

The aim of this study was to find methods which can be used to measure early damage to kidney function and find out how colic affect horses' kidneys by investigating the release of MMP-9 complexes, proMMP-9, MMP-9, proMMP-2 and MMP-2, proteins, AP and GGT into urine with horses that underwent an acute colic operation and by comparing the data with urine and serum creatinine levels and plasma MMP activities. We also wanted to investigate whether a commercially available human cystatin-C test can be used with equine patients.

## Materials and methods

### Selection of cases

Five horses (2 mares, 3 geldings) that underwent a colic operation between September 2002 and November 2003 were included. Ages ranged from 2 to 11 years (mean 7.8 y) and weights, from 450 kg to 540 kg. All the colic horses had received flunixin 1.1 mg/kg before coming to the clinic. Some of the horses had also received benzylpenicillin 10000 – 15000 IU/kg, B-vitamin, detomidine, xylazine or fluid therapy before admission to the clinic. Clinical signs varied from mild discomfort to heavy sweating and restlessness. Three of the horses had been painful for about 12 hours before the operation started (small intestine volvulus, right dorsal displacement and left dorsal displacement), one horse for 24 hours (with small intestine impaction) and one horse had been colicky for 72 hours before the operation (right dorsal displacement). Neither of the mares was pregnant.

Before anesthesia all the horses with colic were given benzylpenicillin 40000 IU/kg and gentamicin 6.6 mg/kg i.v. For sedation, some of the horses received acepromazine and then detomidine (5–20 μg/kg). The more painful horses were also given butorphanol for analgesia. Right before induction, guaifenesin (50 mg/kg) was given, and induction was performed with thiopental bolus.

The control group consisted of seven healthy stallions that came for elective surgery (castration). Ages ranged from 1 to 10 years (mean 5.3 y) and weights from 130 kg to 565 kg (mean 395 kg). The study's protocol was approved by the local ethical committee, and informed owner consent was obtained.

### Experimental design and conditions

Blood was collected before anesthesia in all horses, and urine was collected by spontaneous voiding in six of the horses in the castration group and *via *a urinary catheter soon after induction of anesthesia in the rest of the horses. The plain blood samples were kept at room temperature until serum was separated by centrifugation. The blood samples in lithium heparin and the urine samples were kept in iced water until centrifuged (1300 g, 10 min) at 4°C. A part of these samples were kept at 4°C until analyzed in the same day or next morning and the rest was frozen to -70°C. Serum alkaline phosphatase (AP), urea, creatinine (Cr), aspartate-aminotransferase (AST), sorbitol dehydrogenase (SDH), plasma glucose, and creatine kinase (CK) were all measured using routine laboratory methods. Plasma cystatin-C was measured with Dako Cytomation latex immunoassay^® ^(Dako Cytomation, Denmark) based on turbidimetry (particle-enhanced turbidimetric immunoassay, PETIA) using human cystatin-C-specific antibodies. Plasma MMPs were measured using gelatin zymography as described by Sepper et al. [23]. Before zymography, plasma samples were diluted to aqua in relation 1:5 to minimize the covering effect of plasma proteins to white bands.

Urine protein, Cr, AP, and GGT were measured using routine laboratory methods. Urine enzyme activities are given in relation to urinary creatinine concentrations, that is, U-GGT/Cr and U-AP/Cr (IU/mmol). The U-Prot/Cr relation was calculated by multiplying it by 8.84 to convert creatinine μmol/L to mg/L [1]. Urine samples were cultured for bacterial growth, and pH levels were recorded. All urine samples were also analyzed by dipstick (Bayer Multistix^®^) for occult blood and glucose. Proteolytic activity against extra cellular matrix was measured by using SPS-PAGE gelatin zymography. Zymograms were analyzed for total gelatinolytic activity, complex-, proMMP-9, active MMP-9, and MMP-2 forms. The assays were performed essentially as described by Sepper et al. [23]. Urine samples were first mixed in a 2:1 ratio with sample buffer containing Brom Phenol Blue and protein denaturing 6% SDS (pH 6.8), and preincubated for 30 min. Samples were then loaded (15 μl) into the wells of a 10% gelatin gel (G-2625, Sigma Chemicals Co., St Louis, MO, USA) and electrophoresed at 4°C to avoid spontaneous activation. At the same time Bio-Rad prestained SDS-PAGE standards for low range molecular controls were run. The gel was removed and incubated for 60 min in 100 ml of renaturing buffer (2.5% Triton X-100) that was changed every 10 minutes. The gels were then incubated in +37°C for 18 hours and then stained with Coomassie blue as clear bands against blue background. Activity was determined by placing the gels on a scanner (Scanjet 4C/T, Hewlett-Pacard, Palo Alto, Ca, USA) connected to an image analysis and processing system (Cream tm, Kem-En-Tek, Copenhagen, Denmark). Densitometeric results were calculated in area mode (square pixels) after subtraction of background grey values.

### Statistical methods

The results were calculated by SPSS for Windows. The data were analyzed by the one-sample Kolmogorov-Smirnov test for normal distribution. Normally distributed data were analyzed by the Student's *t*-test to compare between groups. Non parametric data, including urine and plasma MMP activities, were analyzed using the Mann-Whitney test to compare between groups. The minimum statistical difference was taken as p < 0.05.

## Results

**Figure 1:**
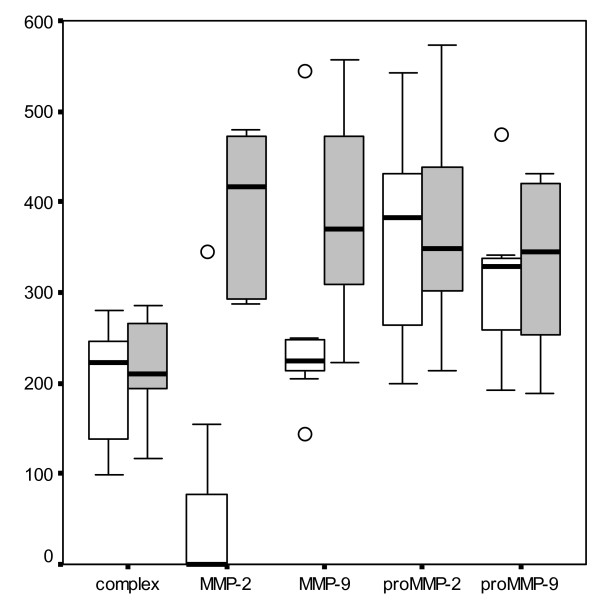
Mean ± s.d. plasma gelatinolytic activity in control group □ and in colic group ■. MMP-9 complex, proMMP-9, MMP-9, proMMP-2 and MMP-2 activities are expressed as an area under the brightness-to-width curve of each gelatinolytic band in zymogram.

Plasma MMP-2 activity was higher in colic horses (p = 0.009) compared to the control group. (Fig. 1)

Mean serum creatinine concentrations were significantly higher in the colic group (p = 0.03), although within reference range in both groups. (Table 1)

**Table 1:** Mean concentrations of serum Cr and glucose in horses with surgical colic (n = 5) and in healthy controls (n = 7).

	**mean ± s.d. (range)**	**Reference values**
**Control S-Cr (μmol/L)**	113.7 ± 15.7 (90.0–135.0)	<168 [34]
**Colic**	146.0 ± 15.9 (126.0–162.0)	
**Control S-Gluc (mmol/L)**	5.7 ± 0.6 (5.0–6.8)	< <8.3–9.7 [27]
**Colic**	11.0 ± 4.1 (6.5–16.6)	
**Colic with glucosuria**	12.7 ± 4.3 (8.1–16.6)	

Plasma cystatin-C concentrations were below the threshold level (<0.39 mg/l) when using human immunoturbidimetric assay.

**Figure 2:**
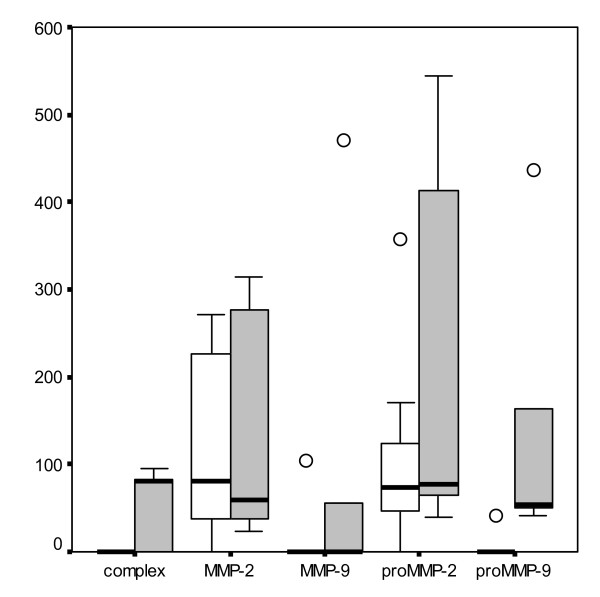
Mean ± s.d. urine gelatinolytic activity in control group □ and in colic group ■. MMP-9 complex, proMMP-9, MMP-9, proMMP-2 and MMP-2 activities are expressed as an area under the brightness-to-width curve of each gelatinolytic band in zymogram.

The activity of urinary proMMP-9 was higher in the colic group (p = 0.03) than in the control group. (Fig. 2) Three out of five colic horses had MMP-9 complexes in urine compared to none in the control group (p = 0.025). Active MMP-9 concentration tended to be higher in the colic horses, although the difference was not statistically significant.

The mean urine protein/creatinine (U-Prot/Cr) ratio was less than 2.0 in control group samples but above it in colic group samples. (Table 2) The difference was not statistically significant.

**Table 2:** Mean concentrations of urine GGT/Cr, AP/Cr (IU/mmol Cr) Prot/Cr (mg/mg) and glucose in horses with surgical colic (n = 5) and in healthy controls (n = 7).

		**mean ± s.d. (range)**	**Reference values for adult horses**
**Control**	**U-Prot/Cr (mg/μmol Cr × 8.84)**	1.06 ± 1.40 (0.33–4.17)	<2.0 [27]
**Colic**		2.17 ± 2.20 (0.67–5.94)	
**Control U-GGT/Cr (IU/mmol)**		1.14 ± 0.53 (0.34–1.94)	1.13 ± 0.54 [31]
**Colic**		5.87 ± 10.61(0.90–24.87)	
**Control U-AP/Cr (IU/mmol)**		1.93 ± 1.17 (0.56–3.6)	2.10 ± 1.45 [33]
**Colic**		3.13 ± 2.17 (1.12–6.45)	
**Control U-Glucose (+drystix)**		0.00	<00 [27]
**Colic**		0.80	

Mean U-GGT/Cr and U-AP/Cr ratios were above reference ranges in the colic group. (Table 2)

U-gluc was negative in all control horses. In the colic group, it was positive in 2 horses of 5. (Table 2)

All urine bacterial growth samples were negative.

## Discussion

The horses with colic in this study had been administered potentially nephrotoxic drugs such as NSAIDs, and gentamicin and they were most probably also endotoxemic before the operation. Matrix metallo proteinases may play a role in the pathogenesis of endotoxemia. Raulo et al. [24] inoculated cows with endotoxin and detected an increase in MMP-2 activity in milk after two hours and an increase in capillary permeability, evidenced first by the penetration of small molecular weight proteins, and 6 to 12 hours post endotoxin inoculation, by neutrophilic leucocytes. Albert et al. [25] showed that in humans plasma activity of MMP-9 increased 30-fold in 2 hours after endotoxin stimulation and fell after 4 to 6 hours. In our study, plasma MMP-2 activity increased in all colic horses. MMP-2 is related to changes in cell permeability which is presumed to happen in colic. Molecules that are smaller than 68 kDa can pass through glomerular filtration. But in most healthy animals the proteins are reabsorbed in the proximal tubules and, thus, very little or no protein is detected in urine samples [1]. ProMMP-2 has a weight of 72 kDa and MMP-2 a weight of 59–62 kDa. Inactive proMMP-9 weighs 92 kDa, but after activation it weighs between 68 and 82 kDa [26]. In our study, urine MMP-9 complexes and both proMMP-9 and MMP-9 activities increased in colic horses which can be related to early tubular damage because these molecules cannot pass through glomerular basement membrane due to high molecular weight. Further studies need to be done to determine the time span over which gelatinolytic activity increases in acute tubular damage.

Unfortunately, the human cystatin-C test did not seem to work in equine patients, which indicates that there is no or very little cross-reactivity between the human cystatin-C specific antibody and equine cystatin-C.

Because no reference values for the U-Prot/Cr ratio for the adult horse could be found in the literature, we followed tentatively Shott's [27] suggestion that values above 2.0 be considered abnormal for horses. In dogs this ratio is considered normal when the U-Prot/Cr is < 0.5 and, borderline between values 0.5–1.0. In dogs with glomerular proteinuria the ratio is >1.0 [27]. In normal 4–5 day old foals Brewer et al. [28] reported a U-Prot/Cr ratio of 0.9 ± 0.5 (1 SD). The mean U-Prot/Cr ratios in the control group were all below 2.0, whereas in the colic group they were elevated when all the horses were included. However, there was one horse with rhabdomyolysis and when that horse was excluded the mean U-Prot/Cr ratio was 1.22, suggesting that no proteinuria indicative to tubular damage could be detected.

None of the control group horses had glucose in their urine, but in the colic group three horses (all the horses that did not eventually survive) had glucose in their urine. Two of these horses had a plasma glucose concentration above the proposed renal threshold of 9.7 mmol/l [27] which can be due to an increase in cortisol or catecholamines. Scheinin and McDonald [29] reported that α2-agonists reduce insulin release from the pancreas and, thus, cause transient hyperglycaemia. On the other hand renal damage to the proximal tubulus cells and their reduced ability to absorb glucose could cause leakage in to urine. Brashier et al. [30] reported glucosuria in ponies with gentamicin-induced nephrotoxicosis. In their study, blood glucose concentrations were below the renal threshold for glucose all the time and glucose appeared in the urine 2–3 days before the S-Cr concentration became abnormally high.

In the study of Adams et al. [31] U-GGT/Cr ratio was 1.13 ± 0.54 IU/mmol Cr for healthy adult horses. Views on what constitutes a clinically significant rise of U-GGT/Cr vary: Kohn and Chew [32] suggested that values above 2.83 IU/mmol (> 25 IU/gCr) were significant, whilst Schott [27] considered a U-GGT/Cr ratio more significant when it is above 11.3 IU/mmol Cr (>100 IU/g Cr) for an adult horse. In our study these enzyme values were clearly below that.

All the mean U-AP/Cr ratios were abnormally high when compared to the "normal" values in the study done by Brobst et al. [22], namely 0.76 ± 0.40 IU/mmolCr, but within normal limits when compared to the study by Edwards et al. [33], namely 2.10 ± 1.45 IU/mmolCr. Schott [25], however, has suggested that a clinically significant rise in U-ALP/Cr is above 3.17 IU/mmol Cr. For dogs, values higher than 20 IU/mmolCr are considered abnormal [20]. In our study the control group horses were all stallions that might have a higher, but still, normal enzyme activity in urine, due to glandular secretions, than geldings or mares. In those samples that were collected in spontaneous urination these secretions could be involved. On the other hand, it may be questionable to compare enzyme activities measured in different laboratories, as there may be differences in the analytical methods.

S-Cr concentration was higher in the colic group, although within reference range. This could be due to reduced renal blood flow because of endotoxemia, or/and NSAID administration and a failure to correct dehydration, because urine enzyme activity was only moderately increased.

Because of the lack of studies it is difficult to determine the threshold values for urine enzymes which in turn makes it difficult to decide whether there is a thread of renal insufficiency that needs to be addressed or transient or reversible kidney damage that does not affect the prognosis of the animal.

The problem with our preliminary study was the small group sizes. However, even with these small group sizes some statistically significant differences between groups could be detected in MMP-activities, even though cell death may have begun, changes in urine enzyme and protein content might occur later on. In many studies where acute kidney damage has been induced by toxic agents, elevated urine enzymes have often been recorded only after several days [16,19,20]. In other studies it has been recorded that urine GGT and AP activities often return to baseline even though S-Cr is still rising, so single samples in the middle of the disease process can give normal values even though kidney damage is progressing [16]. Urine enzyme activities did not correlate in this early phase with either S-Cr concentration or urine MMP activities, which suggests that enzymes enter the urine in bursts rather than in one continuous flow after tubular cell death has occurred.

## Conclusion

The results indicate that plasma MMP-2 may play a role in the pathogenesis of equine colic and urinary MMP-9 in equine kidney damage.

## Competing interests

The author(s) declare that they have no competing interests.

## Authors' contributions

BA participated in performing computer analysis on MMP, analyzing the data and drafting the manuscript. Marja R participated in the design of the study and performed the statistical analysis and helped to draft the manuscript. Minna R participated in supervising MMP analysis. EH and TK collected blood and urine samples and clinical data and helped to draft the manuscript. HS participated in the design of the study. SS carried out other laboratory assays and revised the manuscript.
